# A Hybrid Vacuum Flange RF Oscillator for Low-Cost Mass Spectrometry

**DOI:** 10.1021/jasms.4c00410

**Published:** 2025-01-21

**Authors:** Caraleigh G. Smith, Brian H. Clowers, Steven J. Kregel

**Affiliations:** † Mund-Lagowski Department of Chemistry and Biochemistry, Bradley University, Peoria, Illinois 61625, United States; ‡ Washington State University, Department of Chemistry, Pullman, Washington 99164, United States

## Abstract

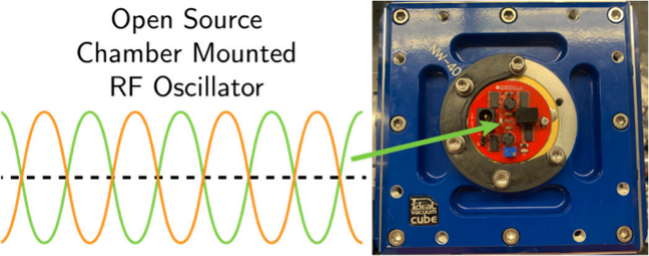

In this communication we report the construction of a printed circuit board which mounts directly to the vacuum chamber of a mass spectrometer and produces the RF waveforms needed by many nonmass-selective devices such as ion guides and ion funnels. Our device is designed to replace a standard KF40 flange, can maintain vacuum chamber pressures of less than 10^–6^ Torr, and contains the circuitry of the open-source Wisconsin Oscillator RF power supply to generate RF waveforms of 1–4 MHz and up to 200 V_p-p_. In this iteration of the Wisconsin Oscillator, we also introduce a variable resistor to control the output RF amplitude and show that its ion transmission capabilities are identical to those provided by commercial RF power supplies. With this new implementation we have greatly reduced the space and monetary requirements for driving nonmass-selective ion manipulation devices, which we expect to be advantageous to those developing low-cost and/or portable mass spectrometry systems.

## Introduction

High voltage radio frequency waveforms are ubiquitous within mass spectrometry to manipulate ions within the vacuum chamber.^1^ The operation of mass analyzers such as quadrupole mass filters and ion traps requires precise control of the RF waveform amplitude and frequency, as these are the parameters which control the mass selective stability of ions within such devices.^2^ However, for devices designed for ion focusing and transport through the vacuum system, such as ion funnels and ion guides, the requirements on the RF waveform are greatly relaxed.^3^ Indeed, such ion transport devices are often designed to minimize mass discrimination and process ions of widely disparate mass to charge ratios. In such cases, the goal is to simply confine the ion within the nonmass-selective device, and sinusoidal RF waveforms of approximately 50–200 V_p-p_ and 1–4 MHz typically suffice.^1^

The generation of sinusoidal RF waveforms for nonmass-selective devices is traditionally achieved by using a large air-core transformer to step up the voltage from a high-power RF source.^4−7^ For example, the transformers utilized in the design put forward by O’Connor are approximately 5 cm in diameter and nearly 12 cm long.^5,6^ Such transformers are not ideal for miniature mass spectrometers, as this arrangement is large and power hungry, limiting the portability and run time of field deployable systems.^8^ With field deployable mass spectrometers in mind, we recently introduced the Wisconsin Oscillator, a small, low-cost, open-source, and self-resonant RF oscillator for mass spectrometry applications.^9^ The Wisconsin Oscillator operates from a low voltage DC power supply, requires less than 1 W of power, and produces two antiphase waveforms of ∼200 V_p-p_ without the use of a transformer. Typical oscillation frequencies for the Wisconsin Oscillator are 1 to 4 MHz depending on the choice of onboard components and the capacitance of the load, and the output can be floated relative to ground through the application of a DC bias. In our previous report we implemented the Wisconsin Oscillator on a custom printed circuit board (PCB) and incorporated that PCB into a stand-alone box, as is typical of home-built electronics. The RF output from this box was then applied to the hexapole ion guides in our vacuum chamber via BNC cables and a standard BNC feedthrough mounted on a KF40 flange.

**Figure 1:**
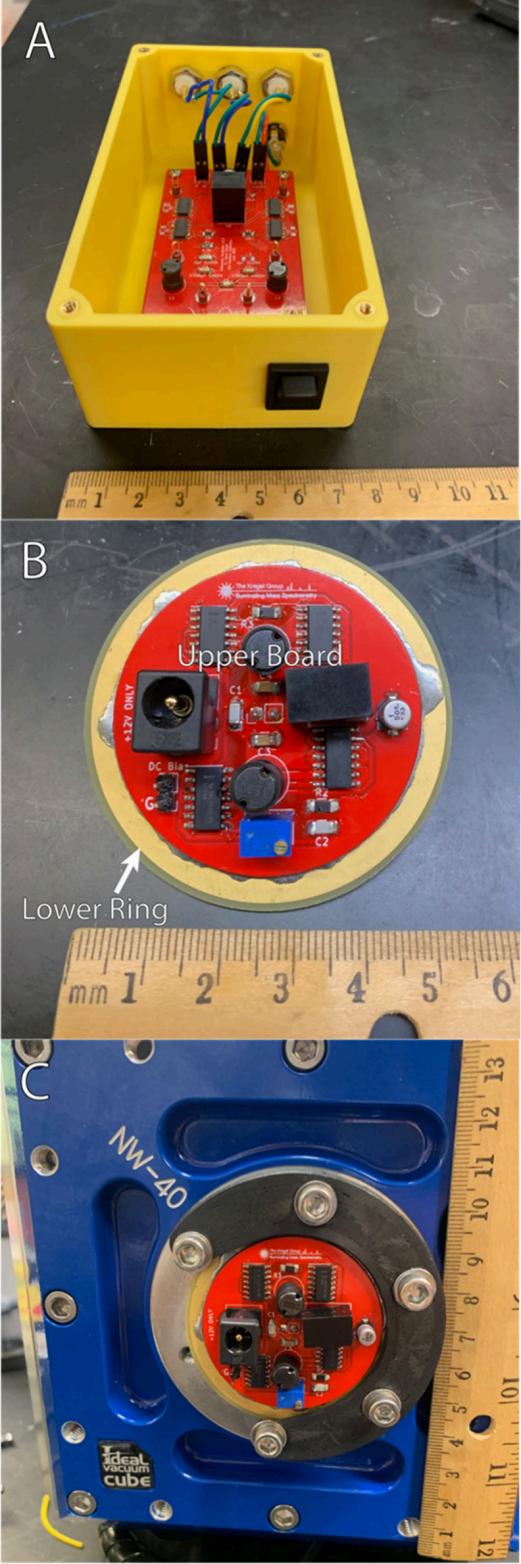
Implementations of the Wisconsin Oscillator. Demarcations on the ruler are in centimeters. A) Original implementation in a standalone enclosure. B) KF40 PCB Wisconsin Oscillator on a benchtop and C) mounted to a vacuum chamber wall.

Electrically, the Wisconsin Oscillator is a large step toward mass spectrometer miniaturization, but the need for a dedicated enclosure, cabling, and vacuum feedthroughs present additional engineering challenges. In this work we reimplement the Wisconsin Oscillator onto a new printed circuit board which is capable of mounting directly to the vacuum chamber housing. Our new design addresses many of the engineering challenges associated with mass spectrometer miniaturization and adds additional capabilities to the Wisconsin Oscillator circuit. As a result of our focus on miniaturization, the entirety of our new implementation occupies significantly less volume than the air-core transformer of previous RF oscillators (see Figure 1).^5,6^ With this new implementation we show ion transmission equivalent to stand-alone OEM oscillators and vacuum levels exceeding what is required for many mass spectrometry applications. Our successful implementation of the Wisconsin Oscillator directly onto the vacuum flange is a step forward for mass spectrometer miniaturization and field deployment and illustrates the possibility of integrating other circuitry directly into the vacuum system.

## Methods

We designed our new Wisconsin Oscillator PCB to mount to a standard KF40 flange. Physically this is accomplished by stacking and soldering two different PCBs together.^10^ The lower ring is a circular board 55 mm in diameter with a 41.25 mm plated through-hole in the center (see Figure 1B). When mounted to the vacuum chamber this lower ring compresses the O-ring and creates the vacuum seal (see Figure 1C). The upper board is soldered to the top of the lower ring and contains all the electrical components needed for the Wisconsin Oscillator. The size of the upper board is dictated by the dimensions of the KF40 flange and mounting clamps; thus, our usable space is confined within a 43 mm diameter circle. The upper and lower boards were joined together using solder paste and a 3D printed jig. When stacked and soldered together the upper and lower boards approximate the shape of a standard KF40 flange and can be mounted directly to the vacuum chamber using standard KF40 O-rings and bulkhead clamps.

**Figure 2:**
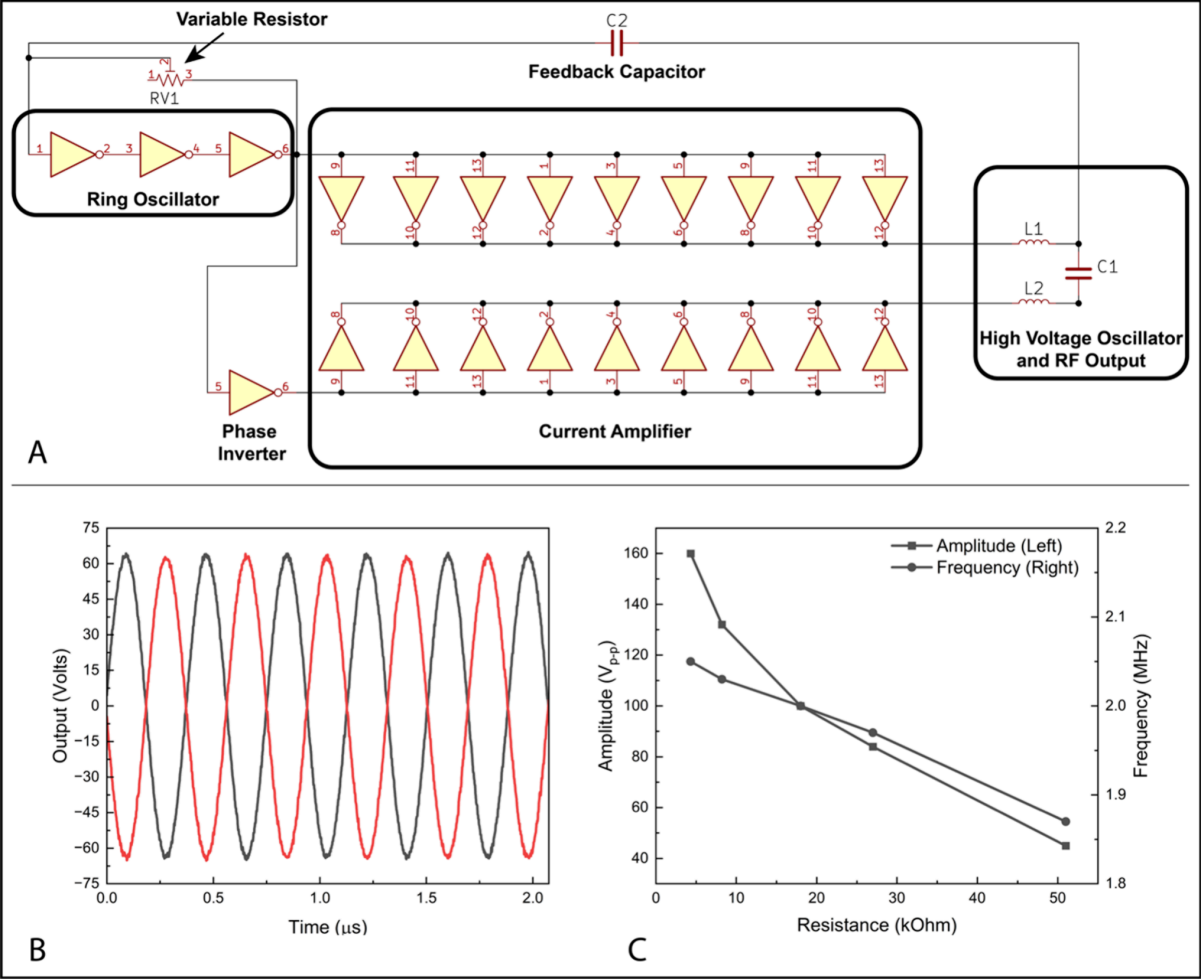
A) Partial electrical schematic of the Wisconsin Oscillator showing the location of the variable resistor used for amplitude control. The RF output is taken from either side of C1. B) Example output waveform from our KF40 oscillator utilizing dual 33 μH inductors to drive a 35 pF capacitive load while the variable resistor was set to ∼8.2 kΩ. C) Data showing the decrease in RF output amplitude and oscillation frequency with increasing values of the variable resistor.

To save space and add functionality on the new PCB we updated some of the components present in the original Wisconsin Oscillator. First, we combined the RF output signals into a single pin header and replaced the power entry pin header with an onboard DC barrel jack connector. We also replaced the single value feedback resistor, R1, with a 50 kΩ trimmer resistor. This trimmer resistor enables the user to vary the output amplitude of the Wisconsin Oscillator and optimize ion transmission through their system (see Figure 2C). We also replaced the original 6.5 V DC buck converter with the Traco Power TSR 1-2465 switching converter due to its identical performance and smaller form factor. With these new components and space constraints in mind, we used KiCAD 7.0 to redesign the Wisconsin Oscillator to fit within the 43 mm diameter circle of the upper board, as dictated by the KF40 vacuum flange. In our new design all the components are mounted to the air side of the PCB vacuum flange for thermal considerations (except for the RF output pins which protrude into the vacuum chamber). Additionally, all the required vias in our circuit are located within the solder pads of the various components. When assembled, the solder used to mount the components also covers the vias and enables vacuum integrity. Assembly instructions and 3D part files are included in the Supporting Information and on our research website: (https://sites.google.com/fsmail.bradley.edu/kregel-group-mass-spectrometry/home/open-source-devlopments). The RF output of our new design is comparable to the output originally reported for the Wisconsin Oscillator.^9^ Depending on the capacitive load and choice of inductors, oscillation frequencies range from ∼800 kHz up to 4 MHz, with a maximum V_p-p_ of approximately 200 V, depending on the specific oscillation frequency. As seen in Figure 2C, the value of the feedback resistor does have a small impact on the oscillation frequency of the circuit, which should be negligible for most nonmass-selective applications.

## Results and Discussion

Our new implementation of the Wisconsin Oscillator into a KF40 compatible form factor produces output waveforms which are functionally equivalent to those in our original design (see Figure 2B). In the original Wisconsin Oscillator^9^ and in our new implementation, the maximum achievable RF amplitude is constrained by the maximum drive voltage of the logical inverter integrated circuits used in the ring oscillator and current amplifier. If higher drive voltage inverter chips become commercially available, it would be trivial to increase the achievable RF amplitude of this circuit.

While higher RF amplitudes are generally desirable, the implementation of a tunable feedback resistor (Figure 2A) in our new design enables the user a degree of amplitude control to optimize ion transmission through their system. As illustrated in Figure 2C, the output RF amplitude decreases significantly with increasing values of the feedback resistor, while the oscillation frequency shows a much smaller dependence. This behavior can be understood by looking at the driving circuitry of the Wisconsin Oscillator in Figure 2A. The high voltage portion of the circuit dictates the output frequency by its resonance condition but is driven by a low voltage ring oscillator. The resonant frequency of the ring oscillator is controlled by the value of the feedback resistor in conjunction with the gate capacitance and output current of the hex inverter chips. Thus, the feedback resistor controls the difference in the resonant frequencies of the ring oscillator and the high voltage output stage, with more similar resonant frequencies leading to higher amplitude outputs. Notably, there is a minimum value for the variable resistor below which no oscillation is observed. As this minimum value is somewhat dependent on the oscillation frequency of the circuit (which itself is a function of the capacitance of the ion guiding device), we have elected not to include a fixed minimum resistance in the feedback circuit. This decision provides each user access to the full amplitude range achievable on their system but does require that the user ensure the circuit is oscillating properly as they adjust the output amplitude. In this work we utilized a variable resistor with a maximum resistance of 50 kΩ to provide a wide range of possible output amplitudes.

**Figure 3:**
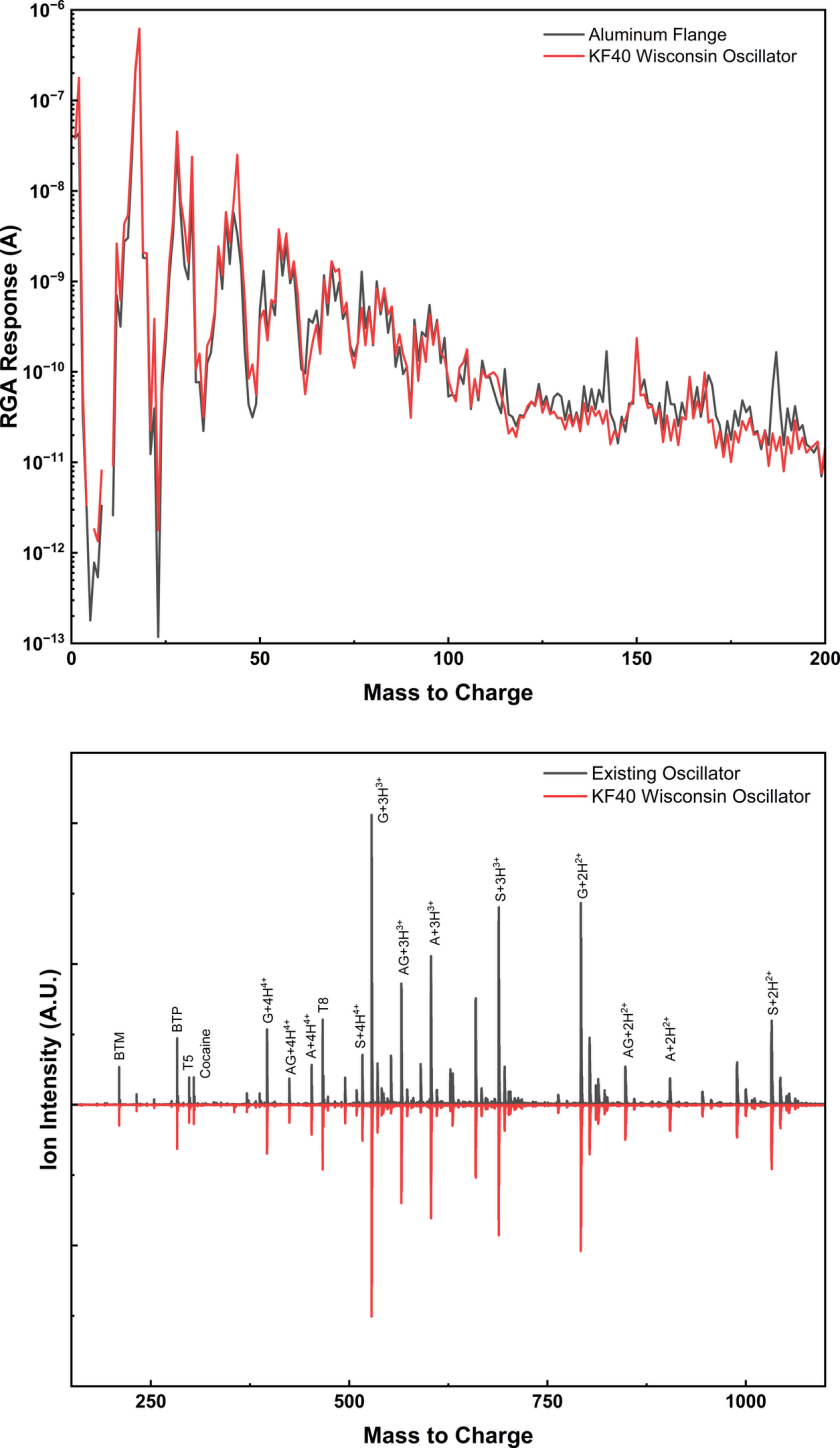
A) Mass spectra showing the residual atmosphere in the vacuum chamber when using a standard aluminum KF40 flange and our new printed circuit board oscillator flange. B) Mass spectra showing ion transmission through a stacked ring ion guide powered by the KF40 Wisconsin Oscillator. The identities of the ions in panel B are included in the Supporting Information.

As our new implementation of the Wisconsin Oscillator is designed to mount directly to the vacuum chamber, we also characterized its ability to act as a vacuum flange. Our circuit was mounted to the KF40 port on one side of a 6-in. vacuum cube from Ideal Vacuum Products, and the chamber was evacuated with a 90 L/s turbomolecular pump backed by a 1.4 L/min diaphragm pump. Initially the pressure in the chamber remained relatively high (∼10 Torr) as residual flux from the solder paste off-gassed into the chamber. However, after a short time, the pressure decreased as expected and the chamber achieved an ultimate pressure of <10^–6^ Torr, equivalent to what we attained using a conventional aluminum KF40 flange. A plot of the chamber pressure as a function of time is included in the Supporting Information. Subsequent venting and re-evacuation of the chamber bypassed the initial off gassing stage. While the chamber was at ultimate pressure, we characterized the makeup of the residual atmosphere with a residual gas analyzer (MKS eVision 2). As seen in Figure 3A, the residual atmosphere was comprised mainly of nitrogen, oxygen, and water as expected. As a result of the presence of only these common gases and the low ultimate pressure achieved, we conclude that any gas present in the chamber is a result of diffusion through the FR4 base of the printed circuit board and the normal leak rate of KF flanges, rather than persistent off gassing from the printed circuit board.

To definitively show that our new implementation of the Wisconsin Oscillator functions as intended, it was used to acquire mass spectra on a Ionicon Analytik T4 time-of-flight mass spectrometer. Our oscillator was mounted to a KF40 port on the vacuum system and connected to a stacked ring ion guide (SRIG) operated at 300 mTorr.^11^ Operating at 1.8 MHz and 80 V_p-p_, our oscillator was used to guide a set of small polypeptide ions from the electrospray ion source into the high vacuum region of the time-of-flight analyzer. As seen in Figure 3B, ion transmission with our new oscillator circuit is nearly equivalent to that provided by the OEM RF oscillator, with the small observed differences accounted for by normal instabilities of the electrospray source.

## Conclusions

Taken as a whole, we expect that our new implementation of the Wisconsin Oscillator will both facilitate the miniaturization of mass spectrometers and help to democratize mass spectrometer instrumentation development. By integrating a low-cost open-source RF oscillator directly onto a standardized KF40 vacuum flange, we have maintained robust ion transmission while greatly reducing the machining and infrastructure requirements (enclosures, cables, etc.) for system development, construction, and modification. Additionally, for field deployable systems, the small size and the low power draw of our new Wisconsin Oscillator (<1 W power draw) will enable lighter and more compact systems to operate independently for longer times. We envision that these advantages will be particularly impactful on systems designed for long-term autonomous operation such as environmental monitoring and interplanetary missions.

## Supporting Information Available

Schematic and fabrication files for the circuit boards used in this work, along with a bill of materials and an instruction guide for assembly (ZIP)
