# Supplementary material for: A Hybrid Vacuum Flange RF Oscillator for Low-Cost Mass Spectrometry
Source: J Am Soc Mass Spectrom. 2025 Jan 21;36(2):236–40. doi: 10.1021/jasms.4c00410 (PMC11808759; doi:10.1021/jasms.4c00410)
Supplement: Supplementary file 1 — js4c00410_si_001.zip [file js4c00410_si_001.zip › KF40 Wisconsin Oscillator SI/KF40 WO SI.docx]

Assembly Instructions for the KF40 Wisconsin Oscillator

This document details the assembly procedures for the KF40 integrated Wisconsin Oscillator circuit. Fabrication files for the two printed circuit boards are included in the Supplemental Information as Gerber files, and a 3D model of the assembly jig is included as a .STEP file. We recommend using a 3D printer to create the assembly jig but machining a metal jig would also be a viable option.

1. Place the upper board face down in the center of the assembly jig and apply solder paste evenly around the back contact ring.


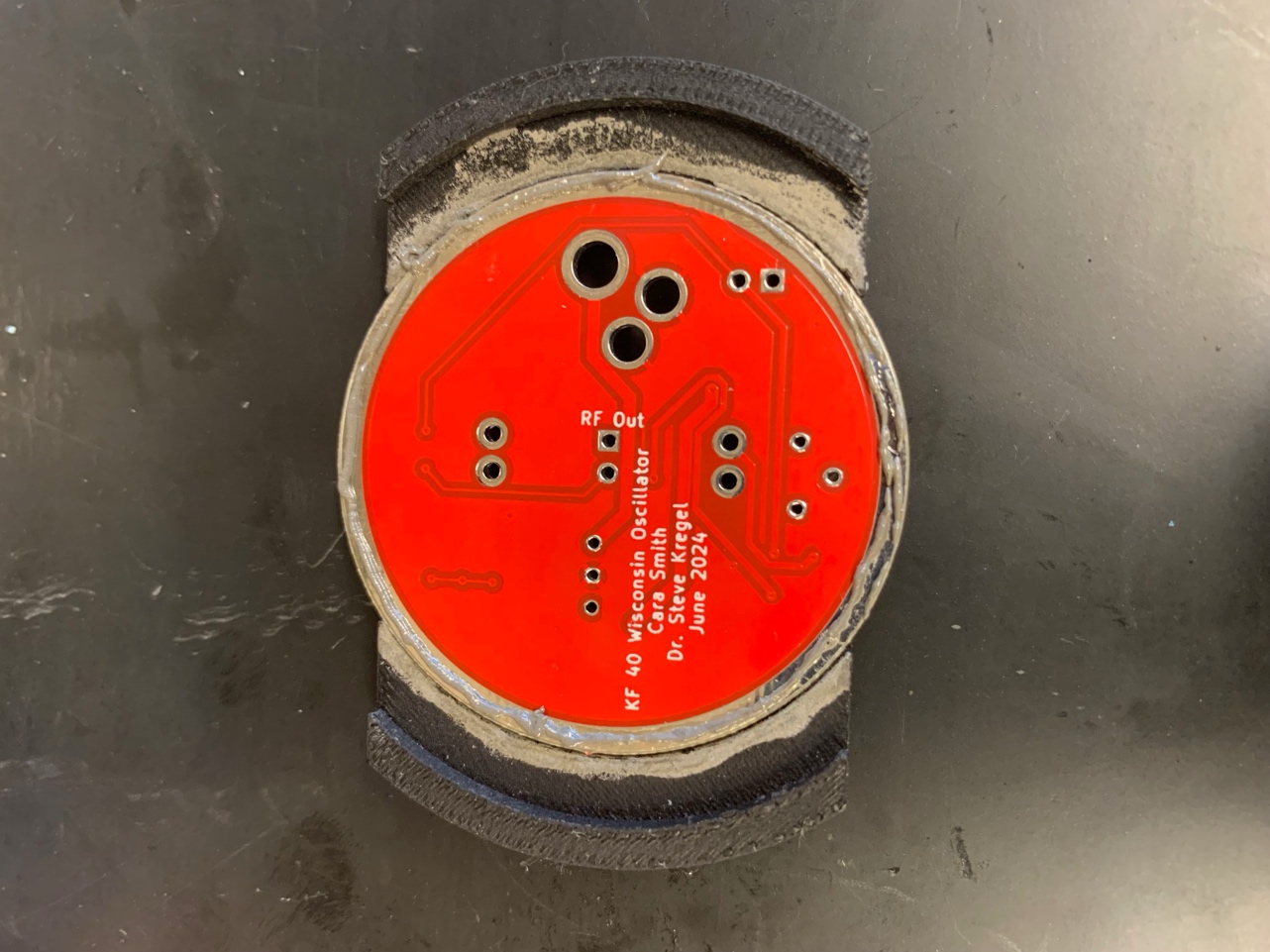


**Figure S1**. The upper ring placed upside down in the assembly jig. Solder paste has been applied around the outside conductive contact for joining to the lower ring.

1. Place the lower ring PCB into the assembly jig and secure with metal binder clips. The clips must be metal as they will hold the assembly together in the toaster oven and will melt if plastic.


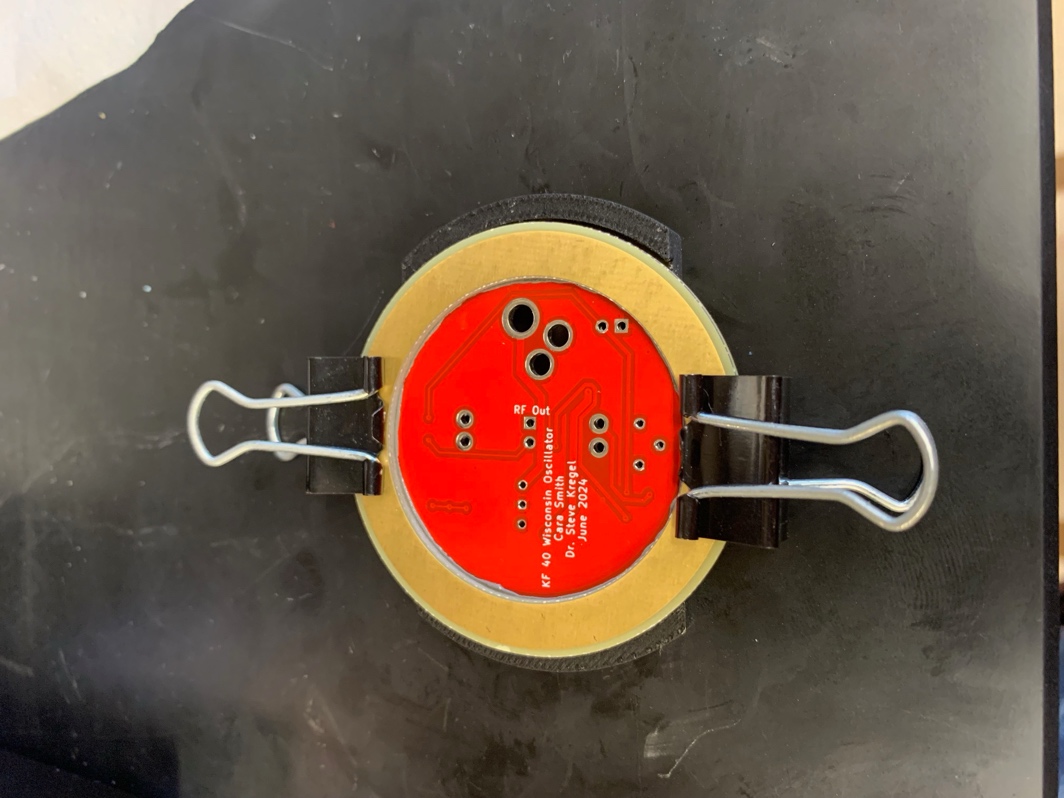


**Figure S2.** The lower ring is placed in the assembly jig on top of the upper ring, and the two PCB’s are secured together with binder clips.

1. Remove the aligned PCB’s from the assembly jig and apply solder paste to the contact pads for U1-U4 as well as the electrolytic capacitor C5.


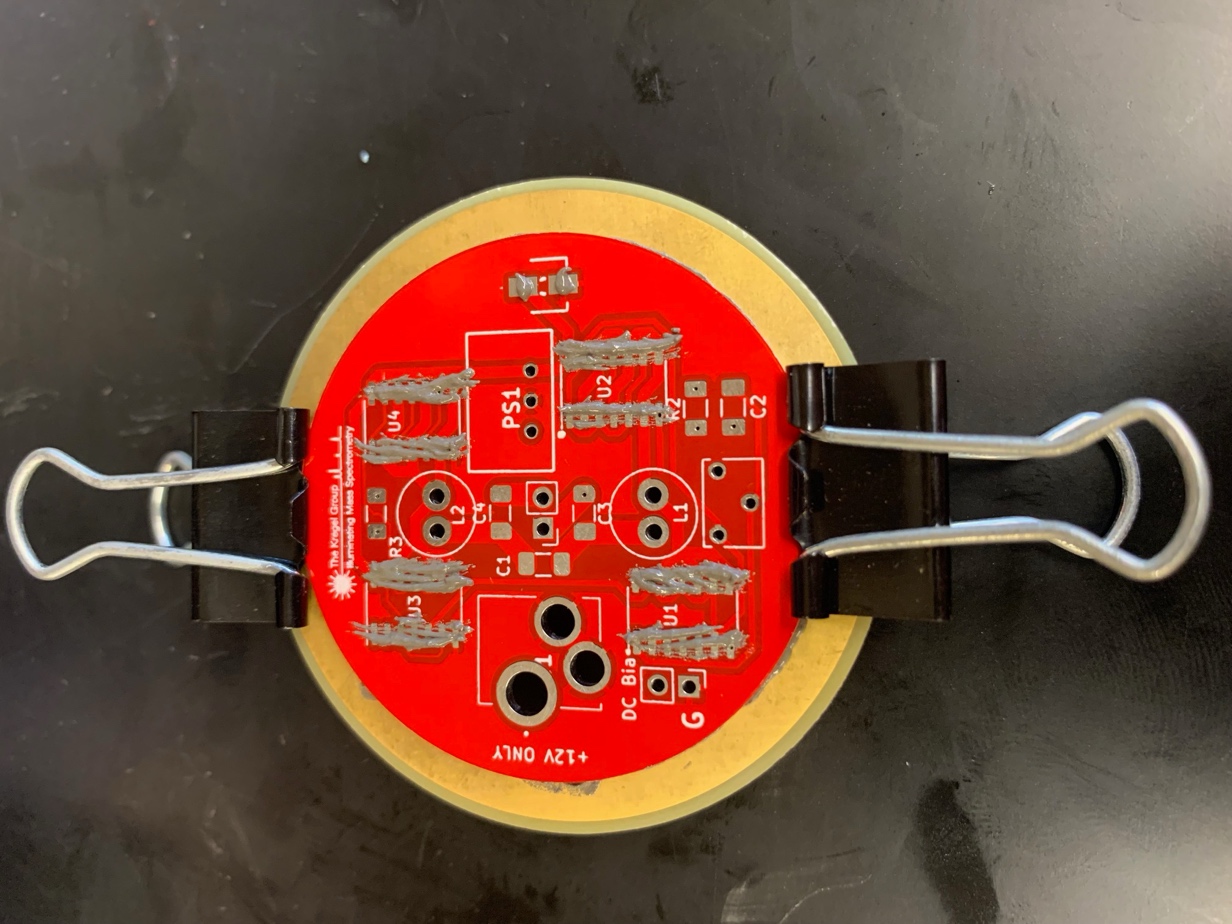


**Figure S3.** The clamped PCB assembly is removed from the assembly jig and solder paste is applied to the connections for U1-U4, as well as C5.

1. Populate U1-U4 and C6 with the appropriate components and place the entire assembly into a toaster oven to melt the solder paste. We use a toaster oven in leu of a proper solder re-flow oven due to the vastly reduced costs. We typically set our toaster oven to ~200 °C on the “air-fry” setting for about 7 minutes, or until the solder paste has melted completely. The entire assembly is then removed from the oven and allowed to cool before removing the metal binder clips. Following this process the oscillator vacuum flange should look similar to the bottom panel of Figure S4. Puddles of solder paste around the edge of the upper board are expected and generally pose no issues for mounting the assembly to the vacuum chamber. Note: it is critical to use this solder re-flow process to properly seal the vias present in the pads of the inverter chips U1-U4.


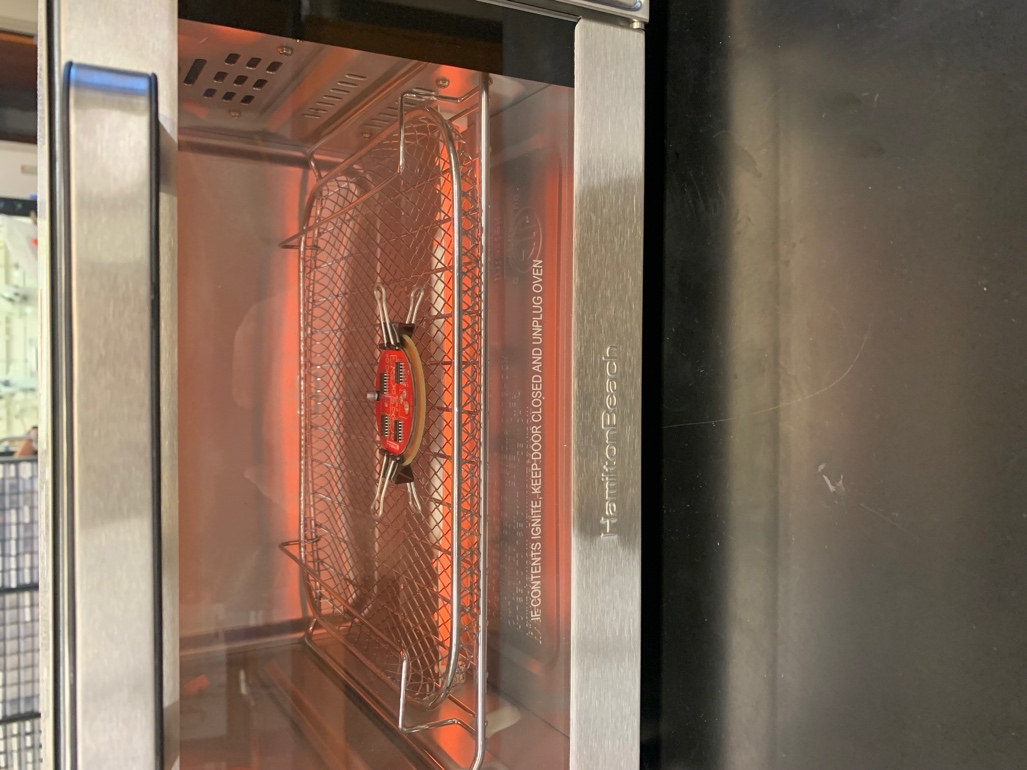

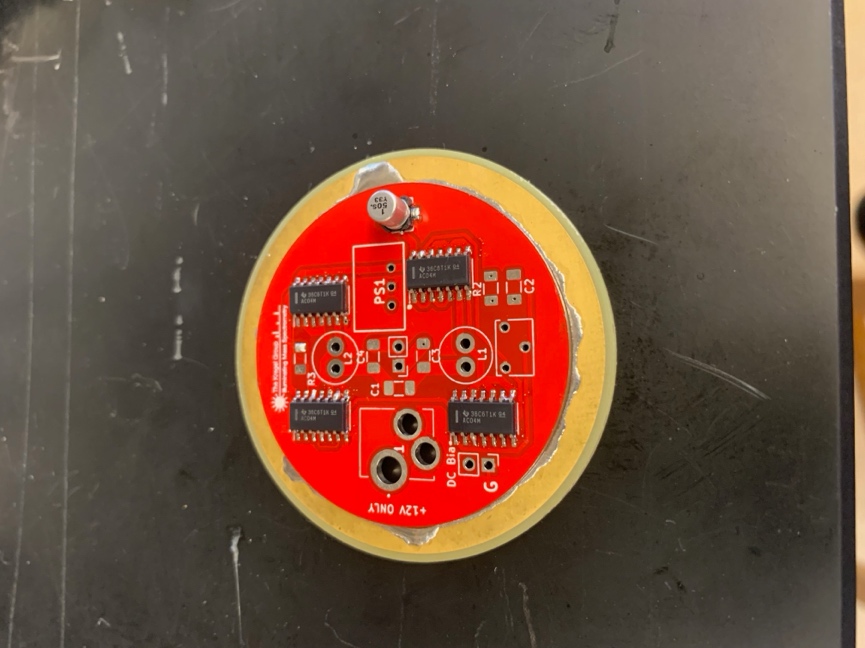


**Figure S4.** Top: U1-U4 and C5 are populated with the appropriate components, and the entire PCB assembly is placed into the toaster oven for reflow soldering. Bottom: The PCB assembly following reflow soldering. Some solder has leaked out from between the two PCBs. A small amount of leakage is acceptable, provided it does not interfere with the bulkhead clamps when mounting the assembly to the vacuum chamber.

1. Solder the remainder of the components onto the upper board. For the surface mount components, this is optionally done in conjunction with step 4 above, but we find it easier to ensure proper placement if done separately. In the toaster oven the assembly rests on the rounded legs of the metal binder clips. This instability tends to tilt the assembly and cause the smaller surface mount components to shift during the re-flow process. Note: the central pin header J1, should be soldered in “backwards” so that the pins extrude into the vacuum chamber when the oscillator flange is mounted to the vacuum chamber.

Lower Ring Board: Compresses KF40 O-ring to create vacuum seal.


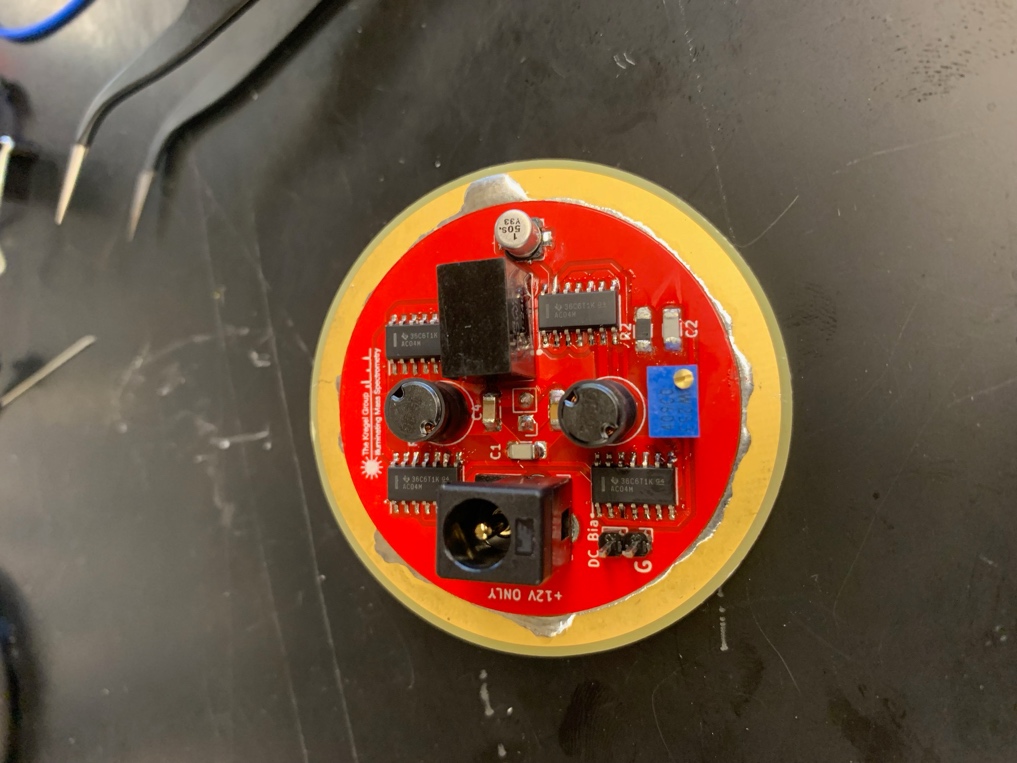

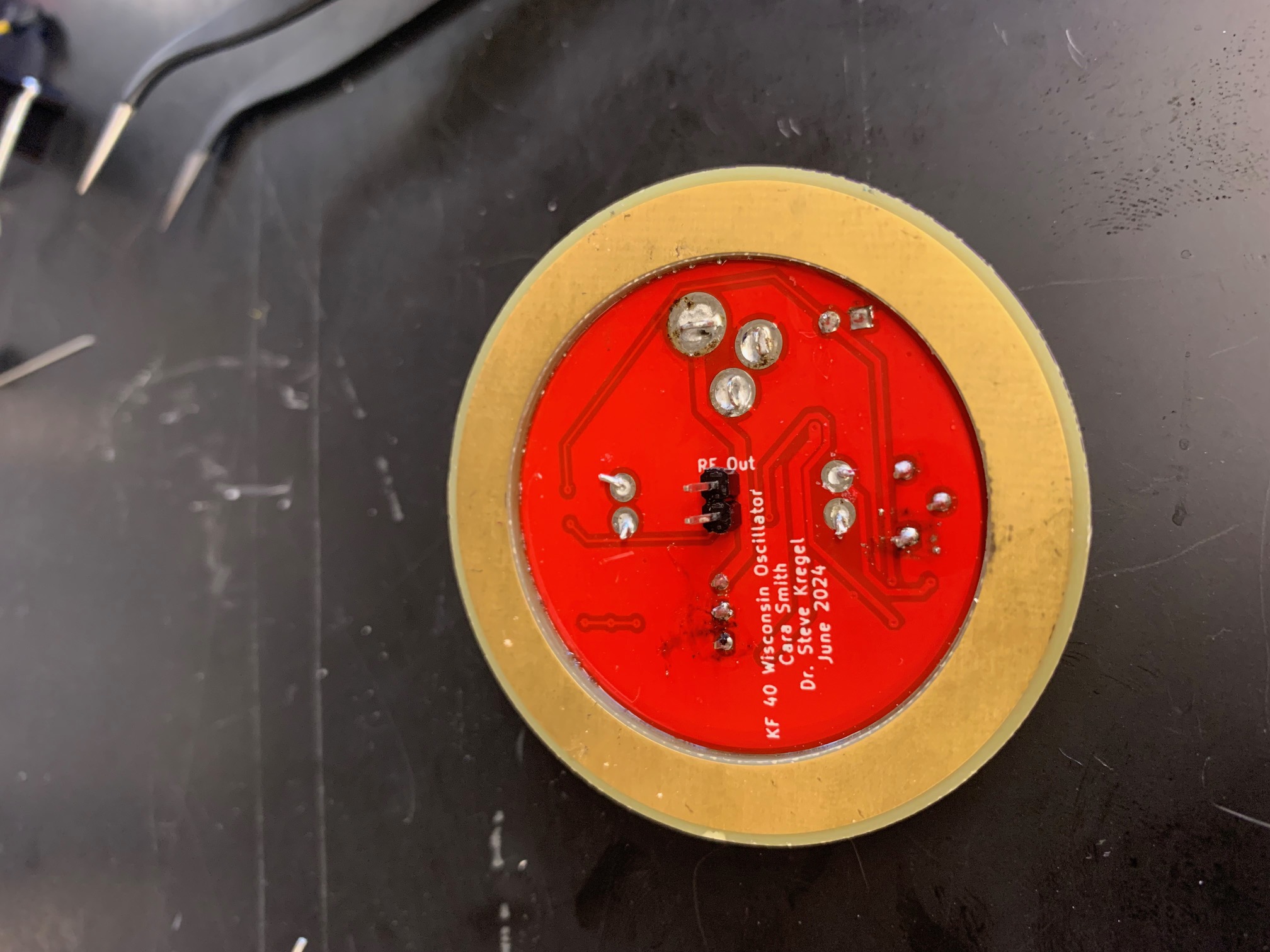


**Figure S5.** Left: Assembled KF40 Wisconsin Oscillator from the air side of the flange. Right: The vacuum side of the same oscillator. Note that the RF output pin header extends into the vacuum for internal electrical connection.


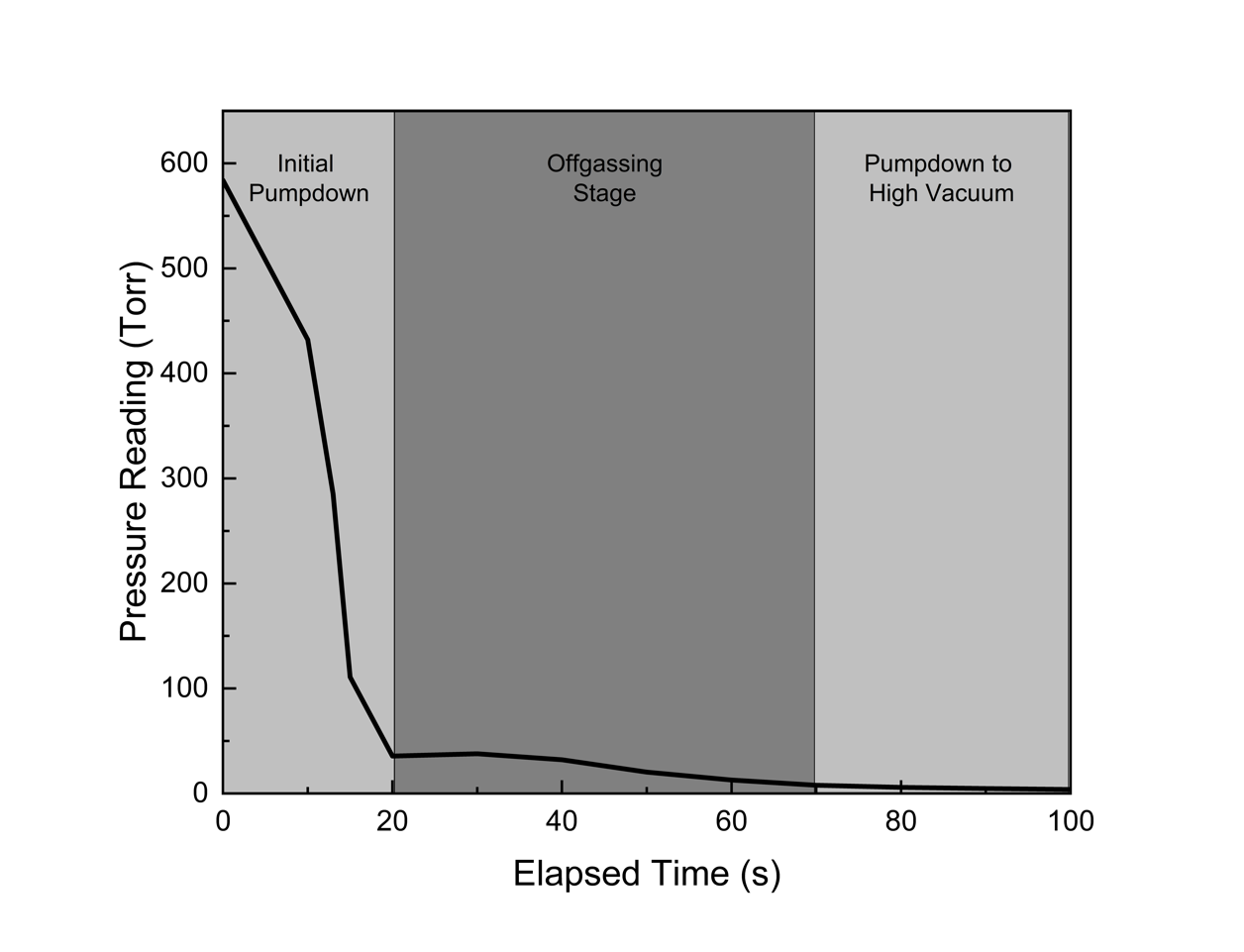


**Figure S6.** Pressure as a function of pumpdown time for a new KF40 Wisconsin Oscillator board. Note the ~50 second plateau from 20s to 70s which arises due to offgassing from soldered components inside the vacuum chamber. This plateau is bypassed during subsequent pumpdowns.

**Table S1.** Identities of the ions in Figure 3B.

| **Label** | **Species** |
| --- | --- |
| G | N-PGGGGKGGGGKGGGGKGGGGK |
| A | N-PAAAAKAAAAKAAAAKAAAAK |
| AG | N-PAAAAKAAAAKGGGGKGGGGK |
| T5 | Tetrapentylammonium |
| T8 | Tetraoctylammonium |
| BTM | Bistrismethane |
| BTP | Bistrispropane |
